# The brain does not process horizontal reflection when attending to vertical reflection, and vice versa

**DOI:** 10.1167/jov.24.3.1

**Published:** 2024-03-01

**Authors:** Alexis D. J. Makin, Giulia Rampone, Marco Bertamini

**Affiliations:** 1 Department of Psychological Sciences, University of Liverpool, Liverpool, United Kingdom; 2 Dipartimento di Psicologia Generale, Università di Padova, Padova, Italy

**Keywords:** symmetry, orientation, sustained posterior negativity, EEG, ERPs

## Abstract

Previous work has found that feature attention can modulate electrophysiological responses to visual symmetry. In the current study, participants observed spatially overlapping clouds of black and white dots. They discriminated vertical symmetry from asymmetry in the target dots (e.g., black or white) and ignored the regularity of the distractor dots (e.g., white or black). We measured an electroencephalography component called the *sustained posterior negativity* (SPN), which is known to be generated by visual symmetry. There were five conditions with different combinations of target and distractor regularity. As well as replicating previous results, we found that an orthogonal axes of reflection in the distractor dots had no effect on SPN amplitude. We conclude that the visual system can processes reflectional symmetry in independent axis-orientation specific channels.

## Introduction

Scientific interest in symmetry perception can be traced to the early observations of Mach (1886), who noticed that reflectional symmetry is more salient than translation or rotation, especially when the axis is vertical. This has since been confirmed with many psychophysical experiments (Barlow & Reeves, 1979; Treder, 2010; Wagemans, 1995; Wenderoth, 1994).

The neural response to visual symmetry was reviewed by Bertamini, Silvanto, Norcia, Makin, and Wagemans (2018). Functional magnetic resonance imaging (fMRI) has shown that visual symmetry activates a network of brain regions in the extrastriate visual cortex. The strongest symmetry activations are in V4 and shape-sensitive lateral occipital complex (Chen, Kao, & Tyler, 2007; Keefe et al., 2018; Kohler, Clarke, Yakovleva, Liu, & Norcia, 2016; Sasaki, Vanduffel, Knutsen, Tyler, & Tootell, 2005; Tyler et al., 2005). This extrastriate symmetry response has recently been replicated in macaque monkeys (Audurier et al., 2022). Symmetry does not activate the striate cortex (V1), where cells with small receptive fields respond to local information. However, V1 may code global axis orientation based on top-down signals (van der Zwan, Leo, Joung, Latimer, & Wenderoth, 1998).

**Figure 1.**
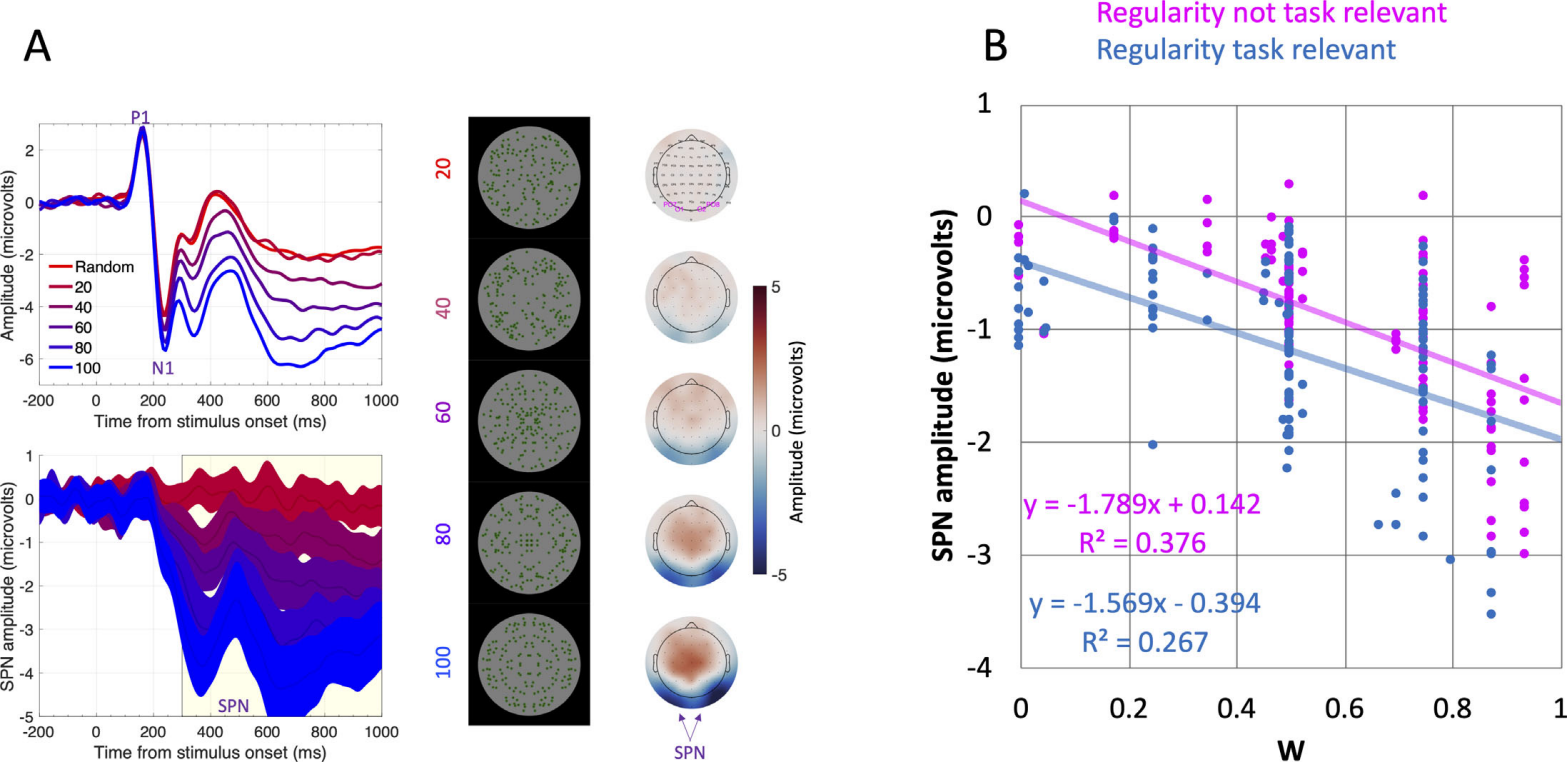
The Sustained Posterior Negativity. (**A**) A typical parametric SPN response from Makin et al. (2020). Top panel shows grand average ERP waves from posterior electrodes. Lower panel shows difference from the random condition with 95% CI ribbons. A large SPN is one that falls a long way below zero. In this example, SPN amplitude increased with the proportion of symmetry in the image. The right panels show the SPN as topographic difference maps, aligned with stimuli. The SPN appears as blue at the back of the head. (**B**) Scatterplot of 227 grand average SPNs from a public database (https://osf.io/2sncj/). “W” on the x axis is a measure of symmetry salience. Blue dots are SPNs from experiments where regularity is task relevant. Pink dots are from experiments where regularity is not task relevant. W and Task both predict SPN amplitude.

The extrastriate symmetry response can be measured with electroencephalography (EEG), as well as fMRI. Symmetrical and asymmetrical stimuli generate an ERP at posterior electrodes. After the P1 and N1 components of the visual evoked potential, amplitude is lower in symmetrical conditions (Jacobsen & Höfel, 2003; Makin, Wilton, Pecchinenda, & Bertamini, 2012; Makin et al., 2016). This symmetry-asymmetry difference wave is called the *sustained posterior negativity* (SPN). A typical SPN is shown in Figure 1A. In this example, SPN amplitude scales with the proportion of symmetry in the symmetry plus noise displays (Makin, Rampone, Morris, & Bertamini, 2020).

The SPN is robust to experimental manipulations of task (Höfel & Jacobsen, 2007; Makin, Rampone, Pecchinenda, & Bertamini, 2013). For instance, Makin et al. (2020) compared five different tasks where participants attended to regularity, color, sound, orientation, or density. A similar SPN response was present in all tasks, although it was selectively enhanced in the regularity task.

Figure 1B shows 227 grand average SPNs from a public repository called the *complete Liverpool SPN catalogue* (Makin et al., 2022). The “W-load” variable on the X axis is a theoretical measure of regularity salience (van der Helm & Leeuwenberg, 1996). The more obvious the regularity, the higher the W-load. SPN amplitude increases (becomes more negative) with W. SPN is also enhanced when regularity is task relevant (blue dots). Figure 1B suggests that no task manipulations can abolish the SPN response to high W regularity (0.6 or above). There is one isolated exception (Rampone, Makin, & Bertamini, 2014), but this was not replicated in a recent unpublished study.

Some previous SPN experiments have manipulated feature attention. Bertamini, Rampone, Tyson-Carr, and Makin (2020) presented mixed patterns with 62 black dots and 62 white dots. One set of dots was task relevant (target), whereas the other could be ignored (distractor). Participants discriminated vertical reflection from random in the target set. There were four combinations of target and distractor. These can be codenamed using Target(Distractor) notation—giving Ref(Ref), Ref(Rand), Rand(Ref), and Rand(Rand). This study produced three SPN difference waves, all computed as the difference from Rand(Rand). If the extrastriate symmetry network was indifferent to distractor regularity, SPN amplitudes would be rank-ordered Ref(Ref) = Ref(Rand) > Rand(Ref) = 0. Conversely, if the network made no distinction between target and distractor, the SPNs would be rank-ordered Ref(Ref) > Ref(Rand) = Rand(Ref) > 0. The observed results lay between these two extremes: Ref(Ref) > Ref(Rand) > Rand(Ref) > 0. Therefore feature attention downweighed the distractor dots but did not suppress them completely.

**Figure 2.**
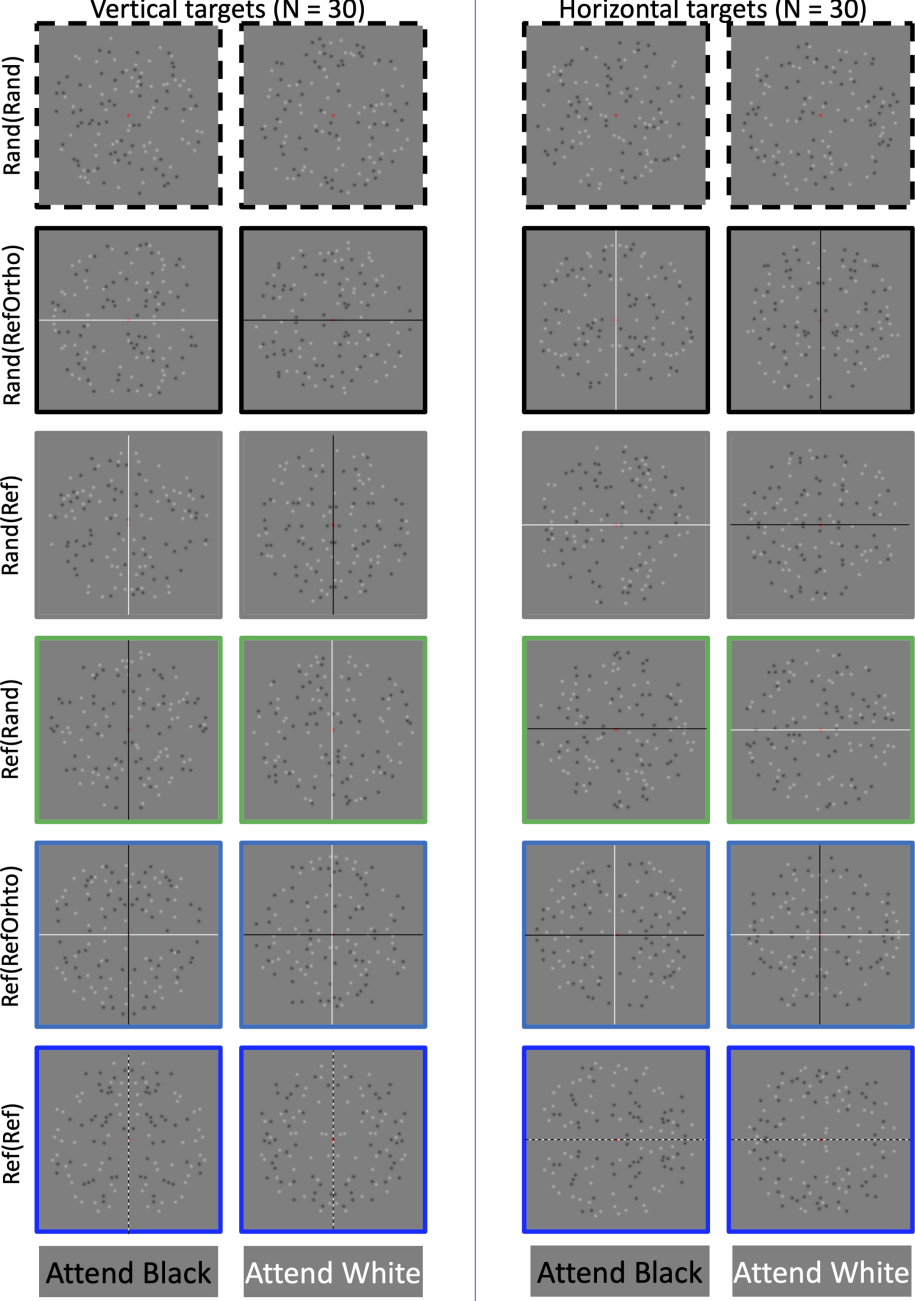
Example stimuli. There were 124 dots, 62 black and 62 white. Participants either attended to the black or white dots. Half the participants classified black(white) dots as symmetry or asymmetry. Colored borders correspond to the colored ERP waves in Figure 3. Horizontal and vertical lines have been added to highlight the axes. These were not present in the experiment.

The current study extended the results of Bertamini et al. (2020). There were 60 participants in total. Unlike in Bertamini et al. (2020), half the participants attended vertical symmetry in the targets (Figure 2 left), and half attended horizontal symmetry in the targets (Figure 2 right). We introduced two new conditions with an independent orthogonal axis of reflection within the distractor dots. These are called Ref(RefOrtho) and Rand(RefOrtho). As in Bertamini et al. (2020), all five SPNs were computed as the difference from Rand(Rand).

The experiment with horizontal targets was completed after peer review of the first experiment with vertical targets. For brevity we combine the results, given that they were very similar in both experiments.

 Although we believe reflectional symmetry processing is very robust, we did not have strong predictions regarding the effect of RefOrtho distractors. Previous literature supports alternative positions. Rainville and Kingdom (2000) found that reflection discrimination thresholds were not elevated by noise masks with different orientations. This suggests that reflectional symmetry is computed independently in separate orientation tuned channels. In support, they found that overlaid spatial frequency masks with different orientations from the target reflection are less disruptive. It is also interesting that reflectional symmetry aids the discovery of objects (Bertamini, Friedenberg, & Kubovy, 1997; Machilsen, Pauwels, & Wagemans, 2009; Wagemans et al., 2012). Independent axes are likely to belong to independent objects, so the visual system may be good at suppressing distractor axes. These observations suggest RefOrtho distractors would not be processed at all, and therefore Ref(Rand) and Ref(RefOrtho) should produce the same SPN. However, SPN priming studies indicate neural overlap between horizontal and vertical reflection (Makin, Tyson-Carr, Derpsch, Rampone, & Bertamini, 2021), and Treder, van der Vloed, and van der Helm (2011) found comparable behavioral results. These observations suggest that task irrelevant RefOrtho may be processed automatically, and therefore Ref(RefOtho) would produce a larger SPN that Ref(Rand).

## Method

Sixty participants were involved (age 18 to 31, mean age 21.3, 11 male, seven left-handed). All participants had normal or corrected-to-normal vision and were reimbursed with course credit. The study had local ethics committee approval and was conducted in accordance with the declaration of Helsinki (revised 2008).

### Apparatus

Participants were seated in a darkened room, 57 cm away from a 53 × 29 cm LCD monitor. This was set to 1920 × 1080 pixels with nominal refresh rate of 60 Hz. EEG data was recorded at 512 Hz using the BioSemi Active-Two system with 64 scalp electrodes arranged according to the extended international 10–20 system. Blinks and eye movements were monitored with external horizontal and vertical bipolar electrodes. These channels were not used in analysis. The experiment was coded in PsychoPy (Peirce, 2007).

### Stimuli

Stimuli were generated using the same code as Bertamini et al. (2020). There were always 124 Gaussian dots within an 11.4° diameter circular region. The size of each dot was 0.46° in diameter, with a gaussian luminance mask with a standard deviation of 1/6 of the diameter. Dot position was constrained so they could not overlap and the minimum distance between centers was 0.5°. White dots had a luminance of approximately 84 cd/m^2^ and black dots of 14 cd/m^2^.

There is a critical perceptual difference between symmetries with two *independent* axes of reflection and those with two *dependent* axes of reflection. The later are sometimes known as double reflection or twofold reflection. According to the bootstrapping model of symmetry perception (Wagemans, van Gool, & D'ydewalle, 1991), the elements of a single-axis vertical reflection are unified into midpoint-collinear virtual trapezoids. The visual system finds additional correlation trapezoids by “bootstrapping” along the vertical global axis. For double reflection, with dependent horizontal and vertical axes, some of these correlation quadrangles are rectangles. Our new Ref(RefOrtho) condition could be called a twofold reflection, if we ignore color, but it does not feature correlation rectangles (Treder et al., 2011).

### Procedure

All participants saw the same stimuli over a total of 408 trials. There were 68 trials in each of the six conditions shown in Figure 2. All participants were presented with the same trials in the same randomized order. This is a slight limitation; however, it is unlikely to have dramatically altered the results. Before the experiment began there was a 12-trial practice block with two repeats of each condition.

Half the participants attended to black dots and half the participants attended to white dots. On each trial the patterns were presented for 1 second, following a 1.25- to 1.75-second fixation interval. The participants then reported whether the dot patterns were symmetrical or asymmetrical using the A and L keys on a standard keyboard. Response mapping was alternated so on half the trials the left key (A) was used to report symmetry and on half the trials the right key (L) was used to report symmetry. Response mapping was not predictable during the stimulus presentation interval.

### EEG analysis

BioSemi Data files were processed with eeglab2022.1 functions in Matlab 2022b. Data from 64 electrodes was ref-referenced to the scalp average, downsampled to 256 Hz, and low pass filtered at 25 Hz, and segmented into −0.5 to 1 second epochs.

Independent components analysis was used to identify artifacts. We used the automated Adjust procedure to remove artifactual components. Between 1/64 and 34/64 components were removed from each participant (*M* = 7.27).

Problematic channels were replaced with spherical interpolation. For 44 participants no problematic channels were identified. In other cases, the number of problematic channels was one (eight participants), two (three participants), three (one participant), four (three participants), or nine (one participant). The channels selected for interpolation were zeroed during ICA cleaning, and then the interpolated signal was reintroduced afterward. This is considered best practice because channel interpolation makes the components nonindependent. After these cleaning operations, trials where amplitude exceeded ±100 microvolts at any electrode were excluded. Average trial exclusion rate was around 8% to 9% in all conditions. On average, 62.8 trials were averaged to produced individual participant ERPs in each condition (minimum 21).

We decided not to remove trials where participants entered an incorrect behavioral response. This would have had two undesirable effects. First, signal quality would have varied between conditions. Second, included trials would not have been representative of their condition. Instead, they would have been a subset where performance superior.

For SPN analysis, we averaged amplitude over a conventional electrode cluster (PO7, O1, O2, and PO8) and a conventional time window (300-1000 ms). These spatiotemporal parameters have been used in many previous SPN studies. For each participant we computed five SPNs as the difference from the Rand(Rand) condition. These five SPNs are termed Ref(Ref), Ref(RefOrtho), Ref(Rand), Rand(Ref) and Rand(RefOrtho).

We also extracted SPNs from the vertical target condition using different conventions to assess vibration of the effect. With this alternative pipeline, downsampling rate was 128 Hz, interpolation was done before ICA, and ICA components were removed manually. The grand average waves from this alternative pipeline were very similar, confirming that our reported results are not problematically dependent on the chosen parameters. The same SPNs were also obtained with smaller and larger posterior electrode clusters (see supplementary analysis on open science framework [https://osf.io/9wn7j/]).

### Statistical analysis

We were interested in confirming the absence of a difference between SPNs in several cases. This is problematic with traditional null hypothesis significance testing. The traditional *p* value gives the probability of obtaining the observed effect (or larger), given the null. However, we are interested in the probability of the null being true given the observed effect. In other words, null hypothesis significance testing gives *p*(D|H0), and we are interested in *p*(H0|D). We thus used Bayesian *t* tests to obtain the desired *p*(H0|D). We computed Bayes factors (*BF*01 and *BF*10) using free JASP software (JASP Team, 2022). We used the default, uninformed prior, which assigns the null and alternative models equal prior odds. With this conventional default in place, *BF*01 = posterior odds in favor of H0, and *BF*10 = posterior odds in favor of H1. We can thus derive *p*(H0|D) with the formula *BF*01/(1 + *BF*01). Bayesian *t* tests also require one to set priors on parameters within the models. We used the default Cauchy prior with an *r* scale of 0.707. *BF*s between 1/3 and 3 are inconclusive. *BF*01 > 3 is evidence in favor of H0. *BF*10 > 3 is evidence in favor of the H1. *BF*01 < 1/3, is evidence in favor of H1. *BF*10 < 1/3 is evidence in favor of H0.

### Sample size considerations

The median sample size in SPN research is 24 (Makin et al., 2022). Our sample of 60 is a large improvement on this. With *N* = 60, we have 80% power for finding within-subjects effects of *d* = 0.36 (*α* = 0.05, two-tailed). Analysis of the SPN catalogue suggests relatively small 0.37 microvolt difference between conditions would typically be associated with an effect of this magnitude. This means our study is powered to find 0.37 microvolt SPNs or SPN modulations. However, the study was completed in two parts, and we observed the results of the vertical target group before running the horizontal target group. The original sample size of 30 was not selected by a priori power analysis, and the second sample of 30 was chosen for consistency with the first.

### Data availability

All aspects of this analysis, presentation scripts, and example stimuli are available on open science framework. This is Project 34 in the complete Liverpool SPN catalogue (https://osf.io/s4n5b/). The recommended analysis pipeline, used for figures and analysis in the manuscript, is in folder called Version 3 2023 Bioline. The alternative pipeline used to assess vibration of the effects in Vertical target group is in a folder called Version 2 2023 Standard Pipeline. The original unpublished analysis is archived in Version 1 2022. Data files and analysis codes used in the current article are available here (https://osf.io/9wn7j/).

## Results

### Behavioral results

**Figure 3.**
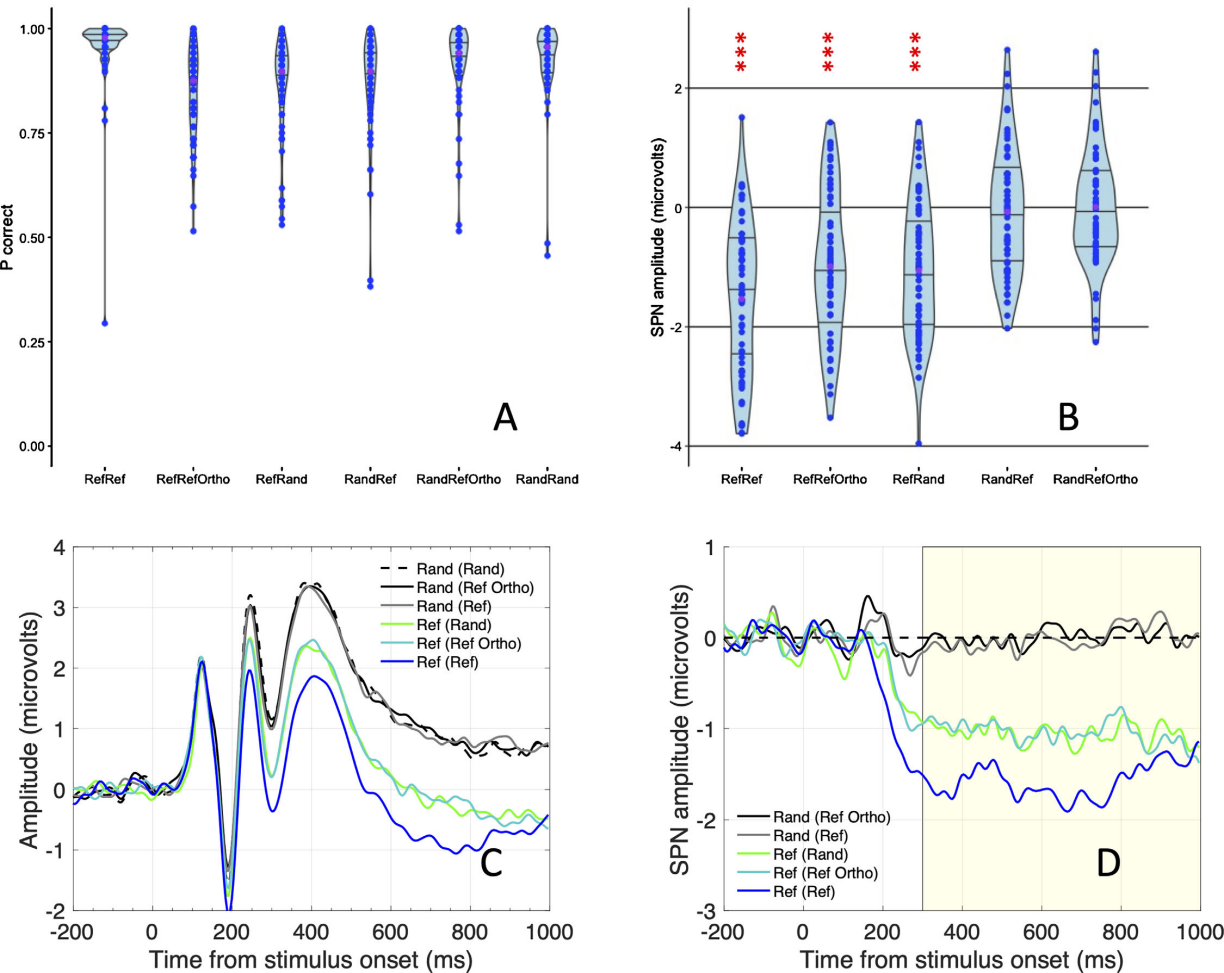
Results. (**A**) Violin plot showing the proportion of correct responses in all 6 conditions. Purple dots are medians. Most participants gave the correct response on most trials. Performance was selectively reduced in the Ref(RefOrtho) and Ref(Rand)conditions. (**B**) SPN amplitudes in five conditions. SPN amplitude was computed as difference from Rand(Rand) in the 300 to 1000 ms window at a posterior electrode cluster [PO7, O1, O2 and PO8]. Purple dots are means. (**C**) Grand average ERP waves from the bilateral posterior electrode cluster. (**D**) SPN waves (as difference from the Rand(Rand) condition). Waves were smoothened with a 5-point moving average filter. ***p < 0.001.

Behavioral results are shown in Figure 3A. Most of the participants were near ceiling. However, a minority were substantially below, and one was at chance levels across all conditions. The *p* correct scores conditions were negatively skewed in all conditions (Shapiro-Wilk test, *p* < 0.01).

Non-parametric Friedman's analysis of variance confirmed that medians differed across the six conditions (*χ*^2^(5) = 104.054, *p* < 0.001). The Ref(Ref), Ref(RefOrtho) and Ref(Rand) conditions are most interesting. Here the correct answer was “symmetry.” Median *p* correct was near ceiling in the Ref(Ref) condition (0.98), but reduced in the Ref(Ortho) condition (0.88) and the Ref(Rand) condition (0.90). The ∼10% performance reductions in Ref(Rand) and Ref(RefOrtho) were significant (*p* < 0.001, Wilcoxon signed ranks tests). Apparently, these task irrelevant distractor dots could not be completely suppressed by feature attention. Averaged across conditions, performance was similar in the vertical and horizontal target groups (0.88 vs. 0.91, *p* = 0.801, Mann-Whitney *U* test).

### EEG results

SPNs from the 5 conditions are shown in Figure 3B, Grand average ERP waves are shown in Figure 3C, and SPN difference waves are shown in Figure 3D. These figures average over the vertical and horizontal target groups.

The SPN was largest in the Ref(Ref) condition. The SPN was reduced, but still present in the Ref(RefOrtho) and Ref(Rand) conditions. The SPN was absent in the Rand(Ref) and Rand(RefOrtho) conditions. None of the 5 SPN distributions violated the assumption of normality (p > 0.077, Shapiro-Wilk test) and there was no violation of the assumption of sphericity (*p* = 0.756, Mauchly's test). A mixed analysis of variance confirmed that there was significant difference among the five conditions (*F*(4, 232) = 39.798, *p* < 0.001, partial *η*^2^ = 0.407). This did not interact with the between subject's factor Target orientation (*F*(4, 232) = 1.837, *p* = 0.123, partial *η*^2^ = 0.031), and there was no main effect of Target orientation (*F* (1, 58) < 1, *NS*).

Pairwise comparisons confirmed the difference between Ref(Ref) and Ref(RefOrtho) (*t*(59) −3.561, *p* < 0.001, *d*_z_ = −0.460) and the difference between Ref(Ref) and Ref(Rand) (*t*(59) = −3.110, *p* = 0.003, *d*_z_ = −0.402). All three conditions where the correct answer was “symmetry” all produced a significantly larger SPN than either of the two conditions where the correct answer was “asymmetry” (smallest effect *t*(59) = 6.271, *p* < 0.001, *d*_z_ = 0.810).

Separate one sample t tests, effect sizes and other statistics for each SPN are shown in Table 1. Table 1 also shows that SPN amplitude never correlated with behavioral performance (largest effect *r* = −0.134, *p* = 0.306).

**Table 1.** These statistics complement the violin plot in Figure 3B. There is additional information about effect sizes (Cohen's d_z_), the proportion of participants from whom the amplitude was < 0 (*p*SPN), the 95% confidence intervals (95% CI). If the mean + 95% CI does not cross zero, we have a significant effect (*p* < 0.05). SPN versus *p* correct shows correlation (*r*) between SPN amplitude and behavioral performance across the 60 participants. The final three rows show *BF*10, *BF*01, and *p*(H0|D).

	Ref (Ref)	RefV (Ref Ortho)	Ref (Rand)	Rand (Ref)	Rand (RefOrtho)
*M*	−1.577	−1.043	−1.054	−0.011	0.008
*SD*	1.312	1.297	1.134	1.166	1.002
Dz	−1.202	−0.805	−0.930	−0.009	0.008
pSPN	0.883	0.733	0.800	0.517	0.517
*t*	−9.308	−6.234	−7.200	−0.070	0.063
*p*	0.000	0.000	0.000	0.944	0.950
*SEM*	0.169	0.167	0.146	0.150	0.129
95% CI	0.339	0.335	0.293	0.301	0.259
minus	−1.916	−1.378	−1.347	−0.312	−0.251
plus	−1.238	−0.709	−0.761	0.291	0.267
SPN vs. *p* correct	0.065	−0.134	−0.077	−0.035	−0.006
*BF*01	<0.001	<0.002	<0.003	7.063	7.066
*BF*10	>1000	>1000	>1000	0.142	0.142
*p*(H0|D)	0.000	0.000	0.000	0.876	0.876

**Figure 4.**
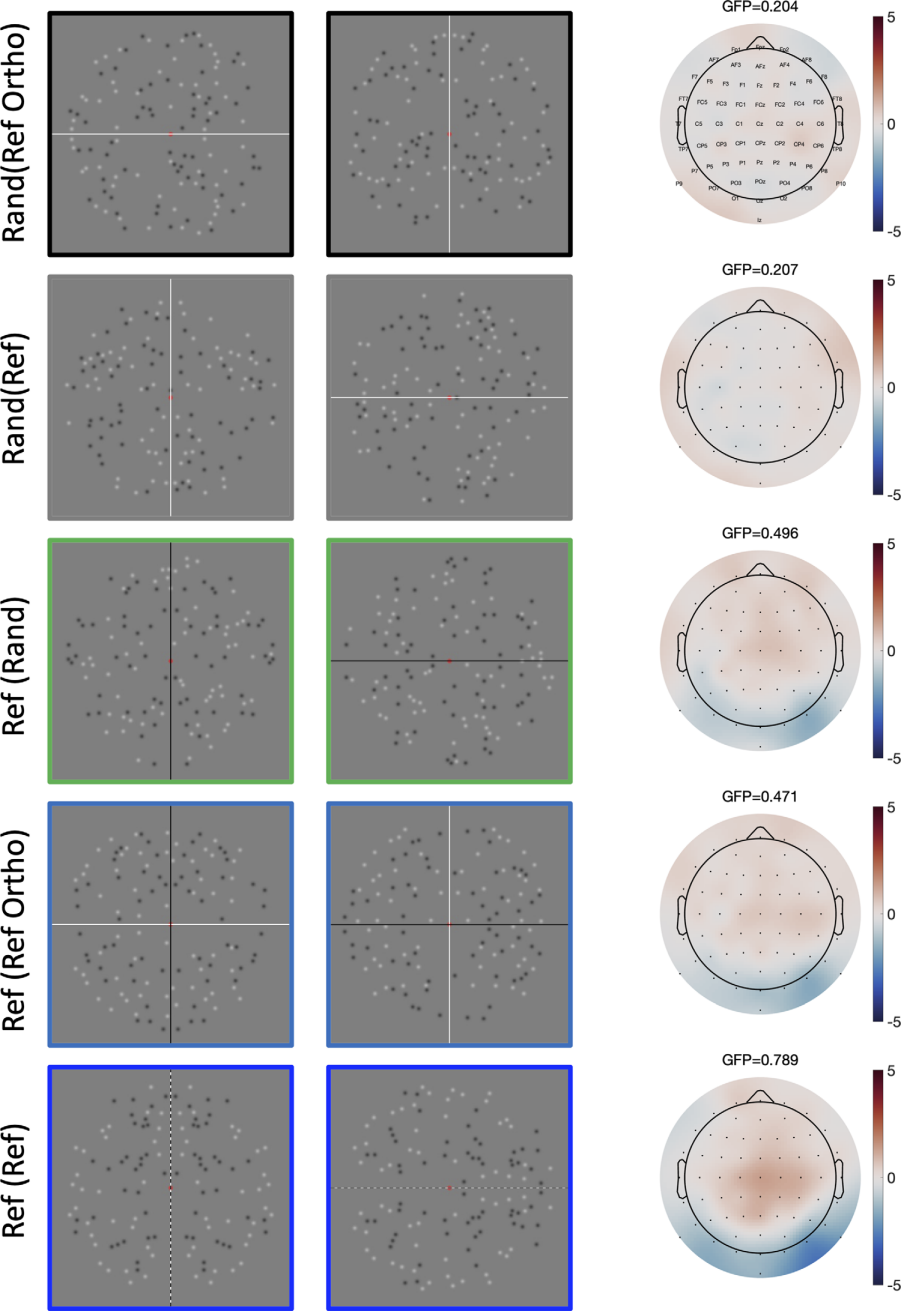
Topographic difference maps from 5 conditions (all compared to Rand(Rand)). Global field power (GFP) is noted above each. Example stimuli are shown alongside as a reminder, with black vertical targets on the left and black horizontal targets on the right.

Topographic difference plots from the 300 to 1000 ms window are shown in Figure 4. The topography was similar in the three conditions that generated an SPN. Figure 4 also shows that global field power of each map increased in line with SPN amplitude (global field power = the standard deviation of amplitudes across the 64 electrodes).

**Figure 5.**
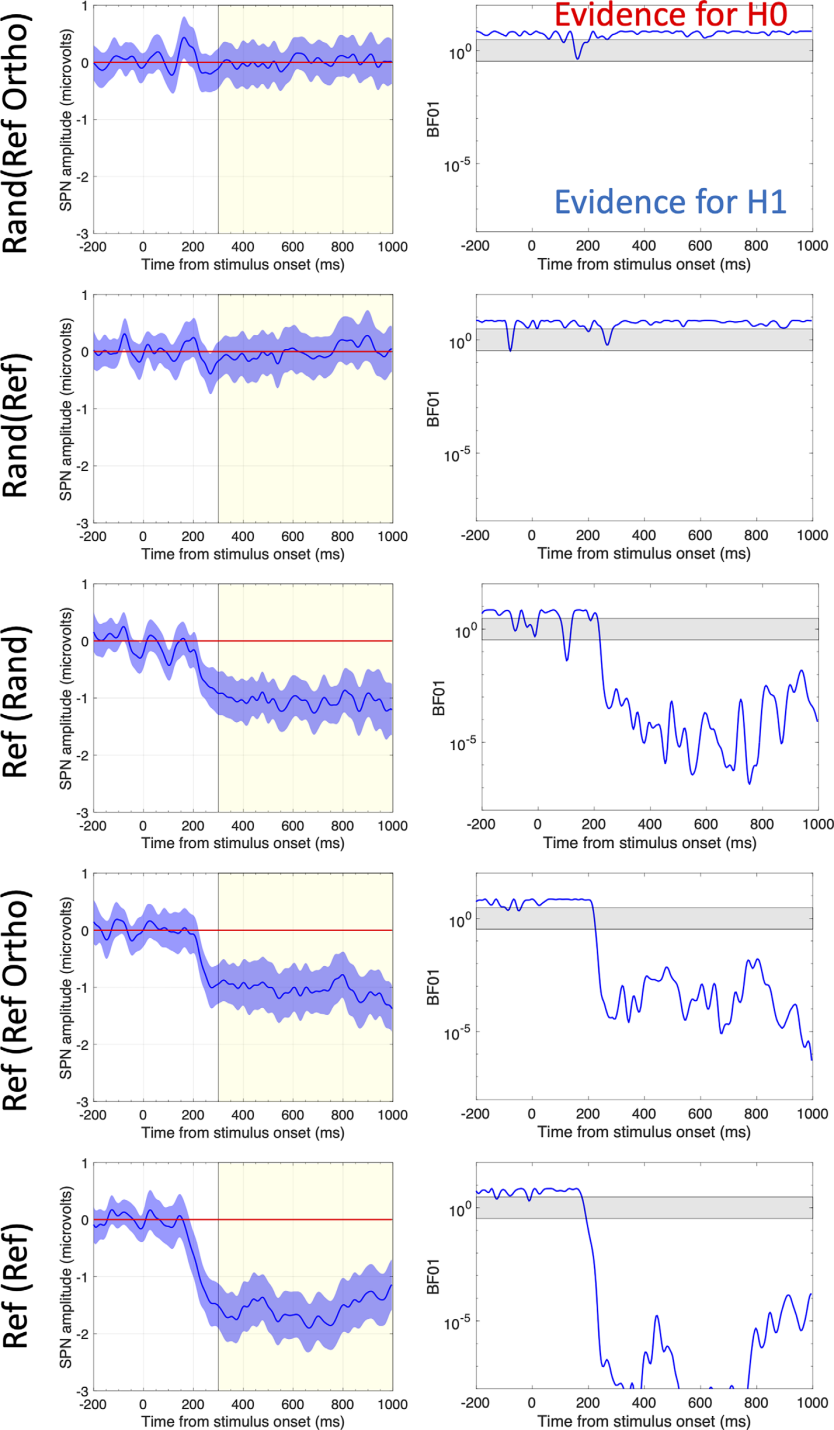
Amplitude vs time and BF01 versus time plots. When the 95% CI does not cross zero, the difference from Rand(Rand) is significant (*p* <0.05). When BF01 > 3, then there unlikely to be a difference from Rand(Rand). When BF01 < 1/3, there is likely to be a difference from Rand(Rand). When BF01 is between 3 and 1/3 (gray band) we remain uncertain.

Figure 5 provides another visualization of the SPN waves. In the left column we have the standard SPN plot, with amplitude in microvolts on the Y axis. The 95% confidence interval ribbons show when significant differences from zero were obtained. The left column shows a *BF*01 wave, where the BF01 from successive Bayesian one sample *t* tests is plotted over time on a log scale. When *BF*01 rises above 3, there is unlikely to be a difference from Rand(Rand). When this falls below 1/3, there is likely to be a difference from Rand(Rand). This suggests no SPN in the Rand(Ref) and Rand(RefOrtho) conditions.

We can also use Bayesian analysis to confirm that the Ref(RefOrtho) and Ref(Rand) conditions produced the same SPN (*BF*01 = 7.065, *p*(H0|D) = 0.876). This again implies that the task irrelevant horizontal reflection was not registered by the visual system when participants were attending to vertical reflection and vice versa.

**Figure 6.**
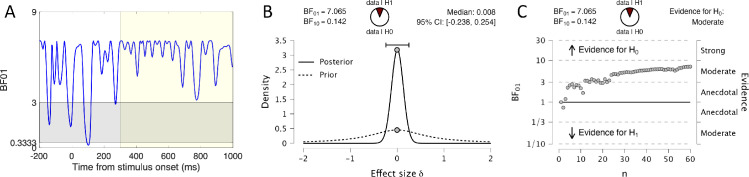
Bayesian analysis confirms that Ref(RefOrtho) and Ref(Rand) produce the same SPN. (**A**) The Ref(Ref) − Ref(Rand) BF01 wave remained consistently above 3 throughout the 300 to 1000 ms SPN interval. (**B**) Prior and posterior plot showing that the null hypothesis (effect size = 0) is 7.065 times more credible than the alternative (effect size not = 0) after observing the data. (**C**) Sequential Bayesian analysis shows that credibility of the null hypothesis increased with sample size.

Figure 6 provides three more visualizations of the crucial Ref(RefOrtho) − Ref(Rand) similarity. Figure 6A shows *BF*01 over time. Figure 6B shows prior and posterior plot from one Bayesian *t* test on amplitude averaged over the 300 to 1000 ms window. Figure 6C shows sequential analysis from this Bayesian *t* test. Figure 6C indicates that *BF*01 drifted further above 1 as more participants were added to the analysis. In summary, Bayesian analysis supports the conclusion that Ref(RefOrtho) and Ref(Rand) waves were the same.

## Discussion

Most previous research has shown that reflectional symmetry is processed automatically, whatever the participant's task. The current work focuses of the case of selective attention to dots with a particular color: black or white. The results indicated that reflectional symmetry with a horizontal axis is not processed at all when participants are attending to reflectional symmetry with a vertical axis. Likewise, reflectional symmetry with a vertical axis is not processed at all when participants are attending to reflectional symmetry with a horizontal axis. This is consistent with the conclusions of Rainville and Kingdom (2000), who claimed that symmetry is computed independently in separate axis orientation-tuned channels. We suggest that attention to symmetry in the vertical channel might block discovery of symmetry in the horizontal channel and vice versa.

Other aspects of the results replicated Bertamini et al. (2020). Feature attention was less-than-perfect. It did not block all interference from distractor dots with the same orientation: Ref(Ref) produced a larger SPN that Ref(Rand). On the other hand, feature attention was better-than-useless: Ref(Rand) produced a larger SPN that Rand(Ref).

Our most important new finding was that Ref(RefOrtho) and Ref(Rand) generate the same SPN. There is an old debate about whether reflectional symmetry is processed preattentively or not (Wagemans, 1995). There is some evidence *against* preattentive symmetry processing. First, reflectional symmetry does not pop out in visual search tasks (Olivers & van der Helm, 1998). Second, reflected contours do not attract spatial attention to regions of crowded displays (Kimchi, Yeshurun, Spehar, & Pirkner, 2016). Third, reflectional symmetry does not produce priming effects in the absence of conscious awareness (Devyatko & Kimchi, 2020). There is also evidence *for* preattentive processing. First, unconscious symmetry processing happens in hemispatial neglect patients, who are subjectively blind the to the left side of objects (Driver, Baylis, & Rafal, 1992). Second, symmetry in task irrelevant outer contours biases judgements about symmetry in task relevant inner contours (van der Helm & Treder, 2009). Third, concentric rings and radial symmetries activate V4 cells in anaesthetized monkeys (Gallant, Connor, Rakshit, Lewis, & van Essen, 1996).

To make sense of these apparent discrepancies, we note that some symmetries are more salient than others. The more salient symmetries are more likely to be processed preattentively. Conversely, a less salient symmetry may only be processed when it is attended. Background reflection with an irrelevant orientation may not be strong enough for automatic preattentive processing. This may change if salience were greatly enhanced, for instance by increasing dot contrast. This is a topic for future research.

Double reflections are more salient than single reflections. They have higher W load (0.75 vs. 0.5), and produce a larger brain response (Makin et al., 2016). It is likely that the second axis of a double reflection *is* processed automatically. When participants are classifying patterns as vertical reflection or random, horizontal plus vertical reflection is classified more rapidly than a single vertical reflection (Palmer & Hemenway, 1978). The current results suggest that the orthogonal axis of two independent axes is ignored, but this does not mean the horizontal axis of a double horizontal plus vertical reflection is routinely ignored.

Van der Helm (2011) revisited the W-loads reported in Nucci and Wagemans (2007). Both articles agree that a vertical reflection has a W-load of 0.5. However, van der Helm (2011) argued that two adjacent vertical reflections have W-load of 0.25, rather than 0.5, because one reflection constitutes noise for the other. If van der Helm's reasoning generalizes to overlapping reflections, then our Ref(RefOrtho) has a W-load of 0.25. This makes it like all other conditions except Ref(Ref), which has a W-load of 0.5. Ref(RefOrtho) and Ref(Rand) both have the same W-load and produce the same SPN. However, despite sharing a W-load of 0.25, Ref(Rand) produced a large 1 microvolt SPN, and Rand(Ref) produced no SPN at all. This highlights the power of feature attention to constrain perceptual organization.

Treder et al. (2011) discussed conditions comparable to our Ref(RefOrtho). They found that this was discriminated better than a single reflection. This apparently contradicts our results. However, the horizontal and vertical reflections were not segregated by color in this study (see Gheorghiu, Kingdom, Remkes, Li, & Rainville, 2016 for more on color segregation and symmetry perception).

Future experiments could experimentally manipulate the orientation difference between target reflection and distractor reflection (e.g., 15°, 30°, and 45°). How different must distractor orientation be before it no longer enhances SPN amplitude? This would provide an orientation tuning function for symmetry perception.

**Figure 7.**
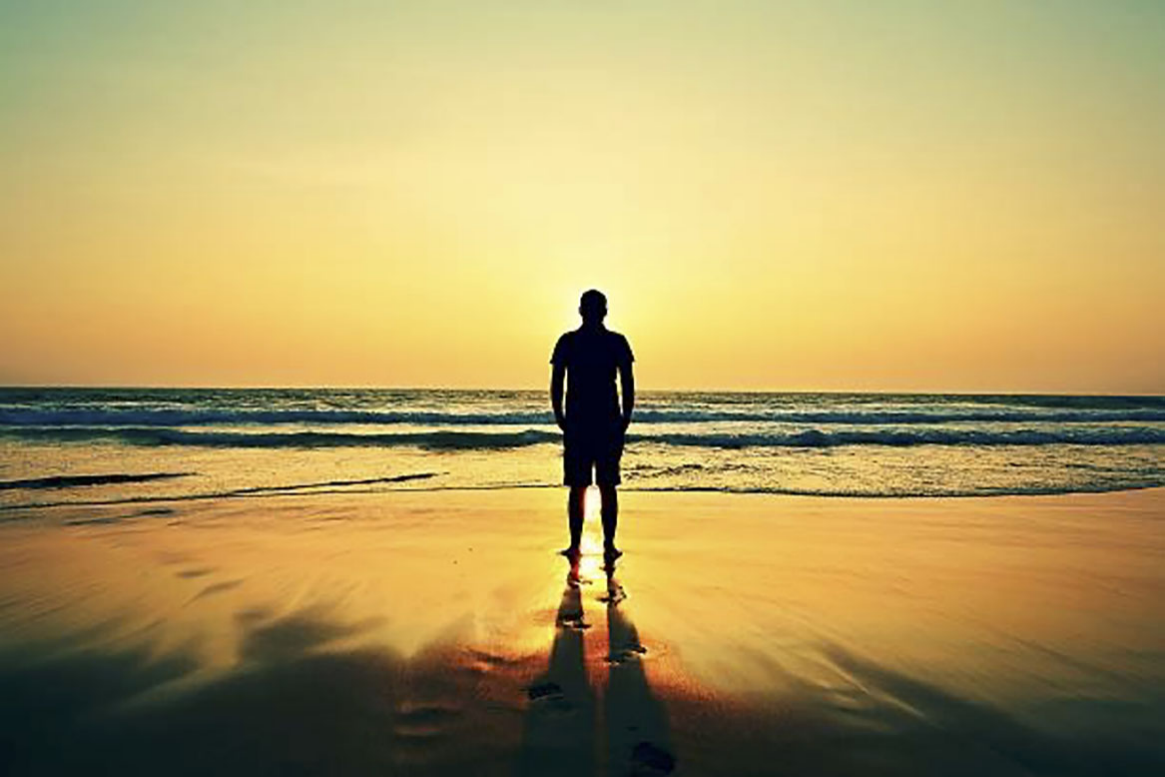
The visual system may ignore the horizon when grouping vertically reflected contours belonging to the man. Image adapted from Google Images (creative commons license).

We finish by noting how our Ref(RefOrtho) stimulus resembles a canonical visual scene, with a vertical symmetrical object occluding the horizon (Figure 7). The visual system may often group vertically reflected contours (caused by a single object or figure), while excluding horizontally reflected contours (caused by the horizontal elements of the landscape). Conversely, the visual system often needs to group reflected elements of different colors if they share the same axis.

## References

[bib1] Audurier, P., Héjja-Brichard, Y., de Castro, V., Kohler, P. J., Norcia, A. M., Durand, J.-B., & Cottereau, B. R. (2022). Symmetry Processing in the Macaque Visual Cortex. *Cerebral Cortex,* 32, 2277–2290, 10.1093/cercor/bhab358. 34617100 PMC9113295

[bib2] Barlow, H. B., & Reeves, B. C. (1979). Versatility and absolute efficiency of detecting mirror symmetry in random dot displays. *Vision Research,* 19, 783–793, 10.1016/0042-6989(79)90154-8. 483597

[bib3] Bertamini, M., Silvanto, J., Norcia, A. M., Makin, A. D. J., & Wagemans, J. (2018). The neural basis of visual symmetry and its role in mid- and high-level visual processing. *Annals of the New York Academy of Sciences,* 1426, 111–126, 10.1111/nyas.13667. 29604083

[bib4] Bertamini, M., Friedenberg, J. D., & Kubovy, M. (1997). Detection of symmetry and perceptual organization: The way a lock-and-key process works. *Acta Psychologica,* 95, 119–140, 10.1016/s0001-6918(96)00038-8. 9062061

[bib5] Bertamini, M., Rampone, G., Tyson-Carr, J., & Makin, A. D. J. (2020). The response to symmetry in extrastriate areas and its time course are modulated by selective attention. *Vision Research,* 177, 68–75, 10.1016/j.visres.2020.09.003. 32987356

[bib6] Chen, C. C., Kao, K. L. C., & Tyler, C. W. (2007). Face configuration processing in the human brain: The role of symmetry. *Cerebral Cortex,* 17, 1423–1432, 10.1093/cercor/bhl054. 16923779

[bib7] Devyatko, D., & Kimchi, R. (2020). Visual awareness is essential for grouping based on mirror symmetry. *Symmetry,* 12, 1872, 10.3390/sym12111872.

[bib8] Driver, J., Baylis, G. C., & Rafal, R. D. (1992). Preserved figure-ground segregation and symmetry perception in visual neglect. *Nature,* 360, 73–75, 10.1038/360073a0. 1436078

[bib9] Gallant, J. L., Connor, C. E., Rakshit, S., Lewis, J. W., & van Essen, D. C. (1996). Neural responses to polar, hyperbolic, and Cartesian gratings in area V4 of the macaque monkey. *Journal of Neurophysiology,* 76, 2718–2739, 10.1152/jn.1996.76.4.2718. 8899641

[bib10] Gheorghiu, E., Kingdom, F. A. A., Remkes, A., Li, H.-C. O., & Rainville, S. (2016). The role of color and attention-to-color in mirror-symmetry perception. *Scientific Reports,* 6, 29287, 10.1038/srep29287. 27404804 PMC4941524

[bib11] Höfel, L., & Jacobsen, T. (2007). Electrophysiological indices of processing aesthetics: Spontaneous or intentional processes? *International Journal of Psychophysiology,* 65, 20–31, 10.1016/j.ijpsycho.2007.02.007. 17400317

[bib12] Jacobsen, T., & Höfel, L. (2003). Descriptive and evaluative judgment processes: Behavioral and electrophysiological indices of processing symmetry and aesthetics. *Cognitive Affective & Behavioral Neuroscience,* 3, 289–299, 10.3758/CABN.3.4.289. 15040549

[bib13] JASP Team. (2022). JASP (Version 0.16.4).

[bib14] Keefe, B. D., Gouws, A. D., Sheldon, A. A., Vernon, R. J. W., Lawrence, S. J. D., McKeefry, D. J., & Morland, A. B. (2018). Emergence of symmetry selectivity in the visual areas of the human brain: fMRI responses to symmetry presented in both frontoparallel and slanted planes. *Human Brain Mapping,* 39, 3813–3826, 10.1002/hbm.24211. 29968956 PMC6175378

[bib15] Kimchi, R., Yeshurun, Y., Spehar, B., & Pirkner, Y. (2016). Perceptual organization, visual attention, and objecthood. *Vision Research,* 126, 34–51, 10.1016/j.visres.2015.07.008. 26440865

[bib16] Kohler, P. J., Clarke, A., Yakovleva, A., Liu, Y., & Norcia, A. M. (2016). Representation of maximally regular textures in human visual cortex. *The Journal of Neuroscience,* 36, 714–729, 10.1523/JNEUROSCI.2962-15.2016. 26791203 PMC6602006

[bib17] Mach, E. (1886). *The analysis of sensations and the relation of the physical to the psychical*. Mineola, NY: Dover Publications.

[bib18] Machilsen, B., Pauwels, M., & Wagemans, J. (2009). The role of vertical mirror symmetry in visual shape detection. *Journal of Vision,* 9, 1–11, 10.1167/9.12.11. 20053102

[bib19] Makin, A. D. J., Rampone, G., Morris, A., & Bertamini, M. (2020). The formation of symmetrical gestalts is task independent, but can be enhanced by active regularity discrimination. *Journal of Cognitive Neuroscience,* 32, 353–366, 10.1162/jocn_a_01485. 31633466

[bib20] Makin, A. D. J., Rampone, G., Pecchinenda, A., & Bertamini, M. (2013). Electrophysiological responses to visuospatial regularity. *Psychophysiology,* 50, 1045–1055, 10.1111/psyp.12082. 23941638

[bib21] Makin, A. D. J., Tyson-Carr, J., Derpsch, Y., Rampone, G., & Bertamini, M. (2021). Electrophysiological priming effects demonstrate independence and overlap of visual regularity representations in the extrastriate cortex. *PLoS ONE,* 16, e0254361, 10.1371/journal.pone.0254361. 34242360 PMC8270198

[bib22] Makin, A. D. J., Tyson-Carr, J., Rampone, G., Derpsch, Y., Wright, D., & Bertamini, M. (2022). Meta Research: Lessons from a catalogue of 6674 brain recordings. *ELife,* 11, e66388. 35703370 10.7554/eLife.66388 PMC9200404

[bib23] Makin, A. D. J., Wilton, M. M., Pecchinenda, A., & Bertamini, M. (2012). Symmetry perception and affective responses: A combined EEG/EMG study. *Neuropsychologia,* 50, 3250–3261, 10.1016/j.neuropsychologia.2012.10.003. 23063934

[bib24] Makin, A. D. J., Wright, D., Rampone, G., Palumbo, L., Guest, M., Sheehan, R., & Bertamini, M. (2016). An electrophysiological index of perceptual goodness. *Cerebral Cortex,* 26, 4416–4434, 10.1093/cercor/bhw255. 27702812 PMC5193141

[bib25] Nucci, M., & Wagemans, J. (2007). Goodness of regularity in dot patterns: Global symmetry, local symmetry, and their interactions. *Perception,* 36, 1305–1319, 10.1068/p5794. 18196698

[bib26] Olivers, C. N. L., & van der Helm, P. A. (1998). Symmetry and selective attention: A dissociation between effortless perception and serial search. *Perception & Psychophysics,* 60, 1101–1116, 10.3758/bf03206161. 9821773

[bib27] Palmer, S. E., & Hemenway, K. (1978). Orientation and symmetry: The effects of multiple, rotational and near symmetries. *Journal of Experimental Psychology-Human Perception and Performance,* 4, 691–702, 10.1037//0096-1523.4.4.691. 722256

[bib28] Peirce, J. W. (2007). PsychoPy - Psychophysics software in Python. *Journal of Neuroscience Methods,* 162, 8–13, 10.1016/j.jneumeth.2006.11.017. 17254636 PMC2018741

[bib29] Rainville, S. J. M., & Kingdom, F. A. A. (2000). The functional role of oriented spatial filters in the perception of mirror symmetry — psychophysics and modeling. *Vision Research,* 40, 2621–2644, 10.1016/S0042-6989(00)00110-3. 10958913

[bib30] Rampone, G., Makin, A. D. J., & Bertamini, M. (2014). Electrophysiological analysis of the affective congruence between pattern regularity and word valence. *Neuropsychologia,* 58, 107–117, 10.1016/j.neuropsychologia.2014.04.005. 24746947

[bib31] Sasaki, Y., Vanduffel, W., Knutsen, T., Tyler, C. W., & Tootell, R. (2005). Symmetry activates extrastriate visual cortex in human and nonhuman primates. *Proceedings of the National Academy of Sciences of the United States of America,* 102, 3159–3163, 10.1073/pnas.0500319102. 15710884 PMC549500

[bib32] Treder, M. S. (2010). Behind the looking glass: A review on human symmetry perception. *Symmetry,* 2, 1510–1543, 10.3390/sym2031510.

[bib33] Treder, M. S., van der Vloed, G., & van der Helm, P. A. (2011). Interactions between constituent single symmetries in multiple symmetry. *Attention, Perception, & Psychophysics,* 73, 1487–1502, 10.3758/s13414-011-0115-9. 21452076

[bib34] Tyler, C. W., Baseler, H. A., Kontsevich, L. L., Likova, L. T., Wade, A. R., & Wandell, B. A. (2005). Predominantly extra-retinotopic cortical response to pattern symmetry. *NeuroImage,* 24, 306–314, 10.1016/j.neuroimage.2004.09.018. 15627573

[bib35] van der Helm, P. A. (2011). The influence of perception on the distribution of multiple symmetries in nature and art. *Symmetry,* 3, 54–71, 10.3390/sym3010054.

[bib36] van der Helm, P. A., & Leeuwenberg, E. L. J. (1996). Goodness of visual regularities: A nontransformational approach. *Psychological Review,* 103, 429–456, 10.1037/0033-295x.103.3.429. 8759043

[bib37] van der Helm, P. A., & Treder, M. S. (2009). Detection of (anti)symmetry and (anti)repetition: Perceptual mechanisms versus cognitive strategies. *Vision Research,* 49, 2754–2763, 10.1016/j.visres.2009.08.015. 19699226

[bib38] van der Zwan, R., Leo, E., Joung, W., Latimer, C., & Wenderoth, P. (1998). Evidence that both area V1 and extrastriate visual cortex contribute to symmetry perception. *Current Biology,* 8, 889–892, 10.1016/S0960-9822(07)00353-3. 9705937

[bib39] Wagemans, J. (1995). Detection of visual symmetries. *Spatial Vision,* 9, 9–32, 10.1163/156856895X00098. 7626549

[bib40] Wagemans, J., Elder, J. H., Kubovy, M., Palmer, S. E., Peterson, M. A., Singh, M., & von der Heydt, R. (2012). A century of Gestalt psychology in visual perception: I. Perceptual grouping and figure–ground organization. *Psychological Bulletin,* 138, 1172–1217, 10.1037/a0029333. 22845751 PMC3482144

[bib41] Wagemans, J., van Gool, L., & D'ydewalle, G. (1991). Detection of symmetry in tachistoscopically presented dot patterns: Effects of multiple axes and skewing. *Perception & Psychophysics,* 50, 413–427, 10.3758/BF03205058. 1788030

[bib42] Wenderoth, P. (1994). The salience of vertical symmetry. *Perception,* 23, 221–236, 10.1068/p230221. 7971101

